# Metabolic aspects of bacterial persisters

**DOI:** 10.3389/fcimb.2014.00148

**Published:** 2014-10-22

**Authors:** Marcel Prax, Ralph Bertram

**Affiliations:** Department of Microbial Genetics, Faculty of Science, Interfaculty Institute of Microbiology and Infection Medicine Tübingen (IMIT), University of TübingenTübingen, Germany

**Keywords:** *Staphylococcus aureus*, persisters, metabolism, toxin-antitoxin system, ppGpp, biofilm

## Abstract

Persister cells form a multi-drug tolerant subpopulation within an isogenic culture of bacteria that are genetically susceptible to antibiotics. Studies with different Gram negative and Gram positive bacteria have identified a large number of genes associated with the persister state. In contrast, the revelation of persister metabolism has only been addressed recently. We here summarize metabolic aspects of persisters, which includes an overview about the bifunctional role of selected carbohydrates as both triggers for the exit from the drug tolerant state and metabolites which persisters feed on. Also alarmones as indicators for starvation have been shown to influence persister levels via different signaling cascades involving the activation of toxin-antitoxin systems and other regulatory factors. Finally, recent data obtained by ^13^C-isotopolog profiling demonstrated an active amino acid anabolism in *Staphylococcus aureus* cultures challenged with high drug concentrations. Understanding the metabolism of persister cells poses challenges but also paves the way for the development of anti-persister compounds.

## Introduction

**Figure 1:**
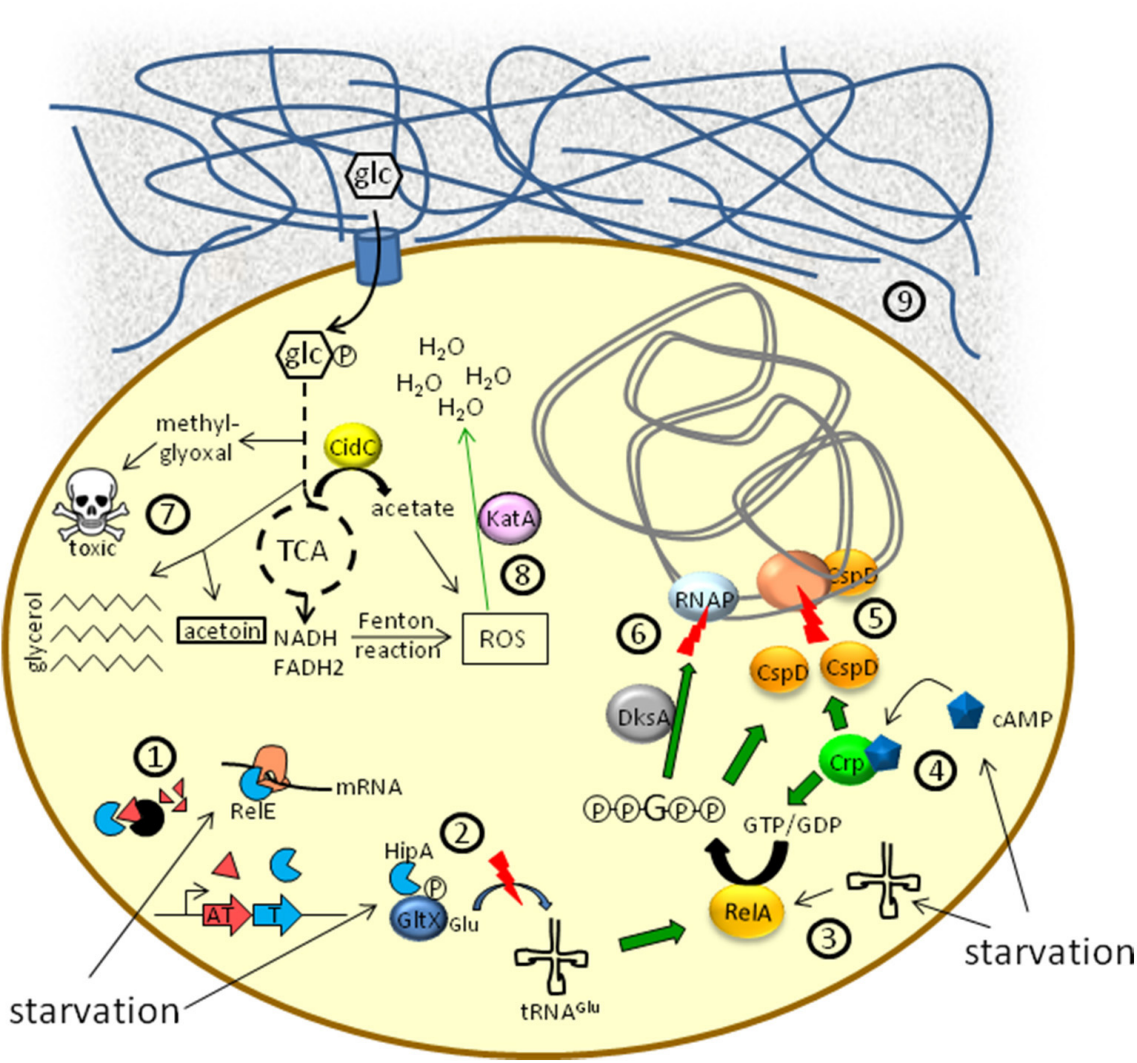
**Schematic overview of metabolic aspects associated with the persister state**. ①, ② Toxin/antitoxin-systems: In a number of bacteria, Lon or Clp proteases, activated in response to starvation, degrade antitoxin proteins ①. Liberated toxins (e.g., RelE) can cleave mRNA or employ a ppGpp-dependent signal transduction ② to induce growth arrest. ③ Uncharged tRNAs due to amino acid starvation lead to the synthesis of ppGpp via RelA. ④ Nutrient limitation favors the synthesis of the second messenger cAMP by adenylate cyclase. cAMP binds to the cAMP receptor protein (Crp) and the cAMP/Crp complex activates the expression of both *relA* and *cspD*. ⑤ Inhibition of DNA replication by CspD. ⑥ Modulation of RNA polymerase (RNAP) activity by the DksA/ppGpp complex. ⑦ Metabolic flux alterations result in a decreased TCA cycle activity and increased persistence. Synthesis of methylglyoxal leads to growth inhibition. Acetoin and triglyceride synthesis represent alternative pathways for the deprivation of pyruvate and acetyl-CoA from the TCA cycle. ⑧ Different branches of metabolism can produce reactive oxygen species (ROS) as hazardous side products, impairing persister formation. Enzymes counteracting ROS activity (e.g., KatA) are upregulated in persister cells. ⑨ Biofilms containing protein- and/or aminosugar-polymer structures (blue meshwork) may represent environments of low supply of nutrients, such as glucose (glc), which favors persister formation.

The treatment of recurrent bacterial infections is often a tedious trial due to antibiotic recalcitrance. This is not solely caused by resistance but also implies persister cells which are (multi-) drug-tolerant (Lewis, 2010). Persisters were first described in 1944 when killing of *S. aureus* with penicillin was found to leave a few survivor cells behind (Bigger, 1944). Notably the antibiotic tolerance of persisters is not genetically manifested, as progenies of persisters are as susceptible as the parent strains (Keren et al., 2004a). Consistent with a number of studies, persisters among an isogenic bacterial culture temporarily reside in a slow- or non-growing state and arise both stochastically and in response to environmental cues. For instance, biofilms accommodate a high level of persisters (Lewis, 2005) and their number within a culture depends greatly on the growth stage, with stationary cultures exhibiting much higher persister levels compared to the exponential phase (Keren et al., 2004a; Lechner et al., 2012). This correlation was confirmed by further studies that established a strong influence between inoculum age and persister frequency (Luidalepp et al., 2011). Retarded protein synthesis as well as protein aggregate accumulation were found to affect the persister levels of a culture (Kwan et al., 2013; Leszczynska et al., 2013), as does bacterial compound signaling (Keller and Surette, 2006). Molecules such as indole, 2′ Amino-acetophenone, or CSP pheromone, some of which are quorum sensing (QS) messengers, can induce drug tolerance and the persister state in different bacteria (Leung and Levesque, 2012; Vega et al., 2012, 2013; Que et al., 2013). Akin to QS, some bacteria have been shown to produce so called resuscitation-promoting factors, converting dormant cells back to a more active state. Among several examples apparently based upon similar mechanisms, the addition of spent culture medium to dormant *S. aureus* cells led to accelerated awakening (Mukamolova et al., 1999; Pinto et al., 2013; Pascoe et al., 2014). The retention of a viable state over longer periods of time and particularly the reversion from dormancy to a growing state requires metabolic activity. One major question is how persisters maintain a critical degree of metabolism over extended periods of time without being killed during hostile conditions. Here, we sum up recent findings on the involvement of metabolism in the persister state (Figure 1) and illustrate the experimental difficulties and challenges accessing this topic. The reader is also referred to a recent review article by Amato et al. (2014).

## Genes linking metabolism and the persister state

The number of identified genes associated with the persister state is steadily increasing and toxin-antitoxin (TA) systems act as key regulators in this regard (Lewis, 2010; Schuster and Bertram, 2013). These systems usually comprise a toxin that blocks or corrupts essential cellular functions and an antitoxin abrogating the toxin's activity. TA systems participate in multiple processes in bacteria, ranging from stress response to regulation of metabolism and survival inside host cells (McKenzie et al., 2012; Helaine et al., 2014). The issue of how TA systems are controlled and how this leads to persister formation has been illuminated in a number of cases. For example, glucose starvation and shortage of amino acids activate RelE-toxin homologs in *E. coli* (Christensen-Dalsgaard et al., 2010). Proteome analysis of starving *Mycobacterium tuberculosis* cells revealed an increased abundance of TA system proteins under nutrient limited conditions (Albrethsen et al., 2013). The alarmone ppGpp is part of the stringent response signaling pathway, which is switched on in response to amino acid depletion. ppGpp abundance and TA system activity appear to be tightly intertwined to control the metabolic state of bacterial cells (Traxler et al., 2008; Bokinsky et al., 2013; Germain et al., 2013; Maisonneuve et al., 2013). For example, the TA toxin HipA phosphorylates the glutamyl-tRNA synthetase GltX which inhibits the loading of tRNA^Glu^ and consequently mimics nutrient limitation resulting in ppGpp synthesis. *Pseudomonas aeruginosa* actively responds to nutrient limitation via a ppGpp-dependent mechanism directing cells to a state of increased antibiotic tolerance (Nguyen et al., 2011). Metabolic stress can also lead to a different scenario, in which ppGpp and the cognate hydrolase SpoT influence persister formation (Amato et al., 2013). Low levels of SpoT thus increase ppGpp abundance that is also associated with DNA gyrase inhibition and reduction of RNA polymerase activity. In *S. aureus* a similar role for ppGpp was demonstrated, as its permanent synthesis leads to growth inhibition and impaired virulence, facilitating persistent infections (Gao et al., 2010). Also the *E. coli* cold shock protein CspD that is expressed during stationary phase and is induced by glucose starvation is influenced by ppGpp (Yamanaka and Inouye, 1997). The lack of nutrients leads to a CspD-dependent inhibition of DNA replication, resulting in increased persister formation (Kim and Wood, 2010). Interestingly, also another second messenger, cyclic AMP (cAMP), is part of the regulatory network of CspD. cAMP, whose physiological level is associated with nutrient availability, increases *cspD* transcription in complex with its receptor protein Crp (Uppal et al., 2014). Moreover, the cAMP-Crp complex also activates the expression of *relA*, resulting in a further increase of the intracellular ppGpp level (Nakagawa et al., 2006). This example illustrates how the metabolic state of a cell can be coupled to persister formation via different pathways to achieve a subtle and precise regulation. In the light of these results, ppGpp seems to be an important mediator between metabolism and persister formation.

Reports about the genetic alterations in the energy metabolism of bacterial cells provide a rather inconsistent picture. *E. coli* mutants lacking *ubiF* or *sucB*, encoding for enzymes involved in ubiquinone biosynthesis, or the TCA cycle, respectively, showed decreased persister levels compared to the wild-type strain (Ma et al., 2010). Both enzymes contribute to the generation of the intracellular ATP pool. However, the inhibition of ATP synthesis by carbonyl cyanide m-chlorophenylhydrazone (CCCP) led to an increased persister formation in another study (Kwan et al., 2013). The same effect was observed for the membrane binding protein TisB of the *tisAB* TA-system. *TisB* expression decreases the proton motive force (PMF) and impedes energy production causing an elevated persister level (Unoson and Wagner, 2008; Dörr et al., 2010).

## Experimental approaches and metabolic peculiarities of persister cells

Major drawbacks in the analysis of persister cells' metabolism are both the natural heterogeneity of the bacterial population and the fact that antibiotics used to isolate persisters destroy their naïve state. Furthermore, the persister state is of temporary nature only and these cells usually merely represent a small subpopulation within a culture. It is therefore of utmost importance to distinguish between results stemming from persister and non-persister cells, which requires efficient means to separate them. Lytic antibiotics or unstable GFP-variants have been used before to address this issue (Keren et al., 2004b; Shah et al., 2006). Obtained transcriptome patterns of suchlike differentiated *M. tuberculosis* or *E. coli* persisters indicated a downregulation of metabolic genes, and therefore a decreased metabolism in persisters (Shah et al., 2006; Keren et al., 2011). Different approaches have been taken to examine the metabolism of persisters more directly. The group of Brynildsen used phenotype microarrays and a fluorescent dye to assay the activity of bacterial reductases as a proxy for metabolic activity (Orman and Brynildsen, 2013a,b). Based on these results, a less active metabolism is apparently not a requirement, but it increases the chance for a cell to enter the persister state. Another powerful technique, termed isotopolog profiling, is based upon feeding of ^13^C-isotope labeled carbohydrates to cultures and subsequent analysis of labeled intermediates. This allows deducing relative activities of metabolic pathways or even networks in a time-resolved manner by comparing ratios of labeled and unlabeled compounds (Eisenreich et al., 2010). Isotopolog profiling provides information of relative metabolic fluxes but not on the quantities or absolute concentrations of metabolites. Measuring the decrease of energy substrates in the medium over time can be theoretically used to determine the metabolic level. We used isotopolog profiling to investigate, which metabolic pathways are active in stationary growth phase *S. aureus* cells that had been challenged with daptomycin (Lechner et al., 2014). *De novo* biosynthesis of amino acids was observed, and their labeling patterns suggested an active glycolysis, TCA cycle and pentose phosphate pathway. Of note, analysis of ^13^C-labeling pattern of Asp and Glu indicated an increased activity of the TCA cycle.

Recent studies provided first insights into the metabolic state of persisters associated with biofilms that provide a protective niche for bacteria against antibiotics and other harmful conditions (Mah and O'Toole, 2001; Donlan and Costerton, 2002; Davenport et al., 2014). This is due, in part, to metabolic downshifts in biofilm dwelling cells. Impaired nutrient penetration and consumption by peripheral cells result in decreased nutrient supply in this environment. Genes involved in TCA cycle and energy production were downregulated in tobramycin challenged and biofilm embedded *Burkholderia cenocepacia* persisters (Van Acker et al., 2013). Metabolic activity can lead to H_2_O_2_ generation by the reduction of molecular oxygen caused by the respiratory chain (Gonzalez-Flecha and Demple, 1995). H_2_O_2_ can thereby accidentally drive the Fe^2+^-dependent Fenton reaction leading to the formation of reactive oxygen species (ROS), which attack essential cellular functions (Imlay et al., 1988). Therefore, long-term survival of a bacterial cell could benefit from an impaired metabolism. In addition, a reduced energy level simultaneously prevents the PMF-dependent uptake of the aminoglycoside tobramycin, as detailed below. In *M. tuberculosis*, redirections in the carbon flux were correlated to growth arrest and antibiotic tolerance (Baek et al., 2011). Acyl-CoA is thereby converted to triglycerides, draining the fuel for the TCA cycle. A further example for increased persistence due to the change of metabolic fluxes is the synthesis of methylglyoxal, which impedes growth of *E. coli* (Girgis et al., 2012). Single deletions of the genes encoding the two metabolic enzymes glycerol-3-phosphate dehydrogenase (*glpD*) or transketolase A (*tktA*) lead to accumulation of dihydroxyacetone phosphate (DHAP) which is finally converted to methylglyoxal. Interestingly, the glyoxylate shunt is upregulated in *B. cenocepacia* persister cells, bypassing NADH production and possible ROS formation via the TCA cycle, illustrating an additional protective mechanism. Another link between persister level and ROS formation in biofilm was established in *P. aeruginosa*, where mutants defective in the stringent response were more susceptible toward antibiotic treatment (Nguyen et al., 2011). Starvation apparently leads to increased antioxidant countermeasures by an upregulation of catalase activity and the restriction of the synthesis of pro-oxidant substances. Furthermore, the metabolic regulator catabolite repression control (Crc) protein decreases the metabolic activity of *P. aeruginosa* in biofilms conferring increased tolerance toward ciprofloxacin (Zhang et al., 2012). These results indicate that a metabolic adaptation process especially in regard to the TCA cycle is involved in the maintenance of the persister state. Besides biofilm cells, the importance of a metabolic downshift was also confirmed by long-term survival assays of planktonic *S. aureus* cells, in which mutants lacking the TCA cycle enzymes aconitase or succinate dehydrogenase showed an enhanced stationary-phase survival level (Somerville et al., 2002; Gaupp et al., 2010). Retarded metabolic flux through or disruption of the TCA cycle was found in clinical *S. epidermidis* isolates with enhanced survival during β-lactam treatment (Thomas et al., 2013). Reduced ROS formation was determined as one critical feature in this regard. In line, ROS activity seems to be involved in programmed cell death, as shown in *S. aureus* (Thomas et al., 2014). ROS formation is apparently linked to acetate production which is again tightly regulated by the two antagonistic factors CidC and AlsSD. CidC is an oxidase converting pyruvate to acetate and is activated by the CidR regulator during the presence of glucose. The CidR regulon also comprises the *alsSD* operon encoding for an α-acetolactate synthase/decarboxylase leading to acetoin synthesis from pyruvate, thereby reducing the amount of acetate by CidC. These data illustrate the strong connection of TCA cycle dependent ROS formation and its negative influence on the long-term survival of cells thereby requiring alternative metabolic pathways to avoid their production.

## Anti-persister strategies and the interplay between carbohydrate supply and persister killing

The importance of persisters in bacterial infections is more and more corroborated (Fauvart et al., 2011). Recent studies indicate that physiology and metabolism could be an Achilles heel for the development of new anti-persister strategies (Allison et al., 2011). A number of compounds counteract the persister state by targeting indispensable cellular processes or by activating resuscitation. These drugs include the acyldepsipeptide ADEP4 which permanently activates Clp proteases or a biphenyl-derivative termed C10 that reverts cells to an antibiotic susceptible state (Kim et al., 2011; Conlon et al., 2013). Manipulating bacterial signaling via artificial QS inhibitors is another approach (Pan and Ren, 2013) and also ppGpp was identified as a potential anti-persister/-biofilm target. A dodecamer peptide termed 1018 was reported to label the alarmone for degradation, thereby inhibiting formation or dispersal of biofilms as sources for recurrent and persistent infections. This peptide was active against at least seven Gram positive or Gram negative bacterial species (de la Fuente-Nunez et al., 2014). In 2011 the group of James J. Collins described that the addition of selected carbohydrates enhanced the killing of persisters by aminoglycoside antibiotics. They established a relationship between the metabolism of selected sugars, the generation of PMF and the enhanced uptake of the drug (Allison et al., 2011). The rate of increased killing is thereby mainly determined by the rate of substrate utilization. Particularly fructose was an effective compound in combination with gentamicin to eradicate *E. coli* as well as *S. aureus* persisters. Subsequent to this finding on metabolite enabled killing, the non-susceptibility of persisters toward aminoglycosides treatment aided in the identification of the most utilizable substrates for such cells (Orman and Brynildsen, 2013b). Another successful approach of metabolite induced killing was demonstrated by combating *P. aeruginosa* biofilms with a combination of mannitol and tobramycin (Barraud et al., 2013) and also arginine and nitrate were described as useful additives in this regard (Borriello et al., 2006). By contrast, an excess of glucose in *S. epidermidis* led to a higher level of dormant cells in a biofilm, presumably due to the accumulation of acidic degradation products resulting from glucose metabolism (Cerca et al., 2011).

## Conclusion

Recent studies highlight the importance of investigating the two interconnected fields of persister state and metabolic activity in bacteria in more detail. Adaptation of the metabolism is a key prerequisite for persisters to cope with hostile conditions. In particular, the modulation of TCA cycle activity appears as a hallmark in persister metabolism. This regulation must be precisely controlled to avoid ROS formation with potentially destructive implications for persister cells. Multiple lines of evidence suggest that the metabolism of persisters can be tuned to alter their susceptibility toward antibiotics or to trigger programmed cell-death-like processes (Rice and Bayles, 2003). In a number of cases, this is achieved simply by supplementing selected carbohydrates. Based upon these findings, new effective anti-persister therapies could be developed to reduce the risk of relapsing or chronic infections. This could result in the development of concerted combination therapies, exploiting the natural metabolic activity of persister cells.

### Conflict of interest statement

The authors declare that the research was conducted in the absence of any commercial or financial relationships that could be construed as a potential conflict of interest.
